# Anti-Cancer Potential of Afzelin towards AGS Gastric Cancer Cells

**DOI:** 10.3390/ph14100973

**Published:** 2021-09-25

**Authors:** Iwona Radziejewska, Katarzyna Supruniuk, Robert Czarnomysy, Kamila Buzun, Anna Bielawska

**Affiliations:** 1 Department of Medical Chemistry, Medical University of Białystok, ul. Mickiewicza 2a, 15-222 Białystok, Poland; katarzyna.supruniuk@umb.edu.pl; 2 Department of Synthesis and Technology of Drugs, Medical University of Białystok, ul. Kilińskiego 1, 15-089 Białystok, Poland; robert.czarnomysy@umb.edu.pl; 3 Department of Biotechnology, Medical University of Białystok, ul. Kilińskiego 1, 15-089 Białystok, Poland; kamila.buzun@umb.edu.pl (K.B.); aniabiel@umb.edu.pl (A.B.)

**Keywords:** afzelin, galectin-3, gastric cancer, glycosylation, MUC1

## Abstract

Afzelin demonstrates anti-inflammatory and anti-cancer properties. Our purpose was to assess its influence on apoptosis, Bax, caspases, MUC1, cancer-related carbohydrate antigens, enzymes participating in their formation, and galectin-3 in AGS gastric cancer cells. A total of 60 and 120 μM afzelin was used in all experiments. Flow cytometry was applied to determine apoptotic response. Western blotting and RT PCR were used to detect the expression of mentioned factors. Flavonoid at higher concentration revealed slight apoptotic respond. *Bax*, *caspase-3*, *-8*, *-9* increased upon afzelin action. Stimulatory effect of the flavonoid on MUC1 cytoplasmic tail and extracellular domain in cell lysates and on *MUC1* gene was revealed. MUC1 release into the culture medium was inhibited by the flavonoid. The 60 μM afzelin dose stimulated GalNAcTL5 protein expression and inhibited C1GalT1. ST6GalNAcT mRNA was inhibited by both flavonoid doses. ST3GalT was inhibited by 120 μM afzelin on protein and mRNA level. Lewis^a/b^ protein was reduced by both afzelin concentrations. FUT3 and FUT4 mRNA was inhibited by 120 μM dose of afzelin. Galectin-3 protein increased in cell lysates and decreased in culture supernatant by 60 and 120 μM flavonoid. *Galectin-3* gene expression was stimulated by two used concentrations of afzelin in comparison to control. We conclude that afzelin can be considered as the potential anti-cancer agent, supporting conventional cancer treatment.

## 1. Introduction

Gastric cancer (GC) is one of the most common malignancies and leading cause of cancer-related death worldwide. Due to a lack of specific signs of early gastric cancer, most patients are diagnosed too late and at advanced stages, with overall poor prognosis [1]. Therefore, looking for new therapeutic strategies, e.g., utilizing anti-tumour products with natural compounds with low by-effects, seems to be very reasonable.

**Figure 1:**
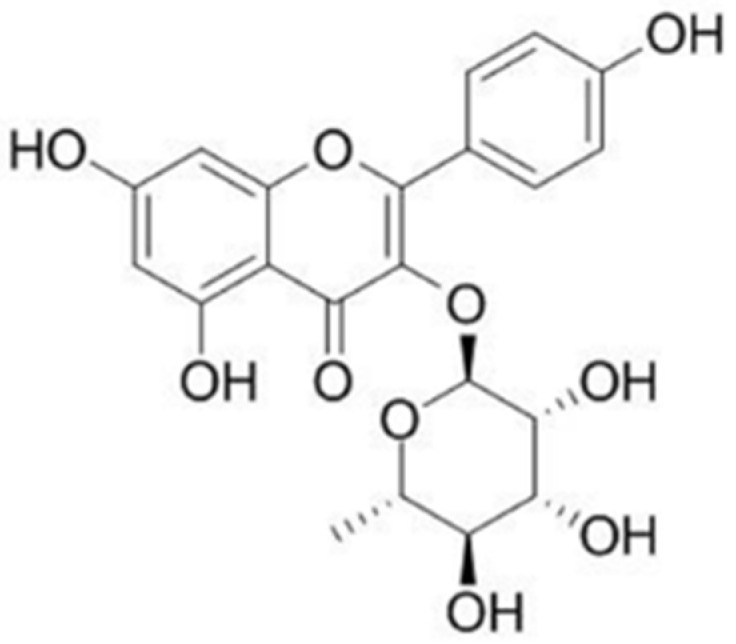
The structure of afzelin.

Medicinal plants are a major origin of potentially therapeutic molecules. Afzelin (kaempherol-3-rhamnoside), a flavonoid found, e.g., in *Nymphaea odorata*, is one of such compounds (Figure 1). It was reported to inhibit the growth of breast cancer cells by stimulating apoptosis [2], suppress cell proliferation in prostate cell lines [3], and demonstrated anti-inflammatory properties [4].

Dysregulation of apoptosis, programmed cell death, is typical feature of cancerous cells, often leading to malignant transformation [5]. Aberrant glycosylation is also considered as the hallmark of cancer and represents one of the most common post-translational modifications occurring during neoplastic modifications. Glycans, as major components of glycoproteins, glycosphingolipids, and proteoglycans, have been shown to be implicated in various steps of tumour development, proliferation, invasion, and metastasis by cell–cell and cell–matrix interactions, cell differentiation, or cancer cell migration [6]. Thus, affecting the specific, cancer related glycan structures seems to be a promising approach with potential anti-cancer treatment applications. One of the main glycans carriers of the gastric epithelium is MUC1 mucin, a transmembrane glycoprotein significantly overexpressed in most adenocarcinomas of epithelial origin. Normally, MUC1 consists of a large, heavily glycosylated extracellular N-terminal subunit, protruding from the cell surface up to 200–500 nm, a transmembrane domain and a short C-terminal cytoplasmic tail. In cancers, mucin losses its apical-basal polarity and becomes distributed over the entire surface of the cell. Cancerous glycoforms of MUC1 differ from glycans of mucin in normal epithelial cells [7].

In most cancers, long and branched glycan forms are truncated and exhibit short Tn (GalNAcα1-O-Ser/Thr) and T (Thomsen-Friedenreich; TF) (Galβ1-3GalNAcα1-O-Ser/Thr) antigens as well as their sialylated forms sTn (Neu5Acα2-6GalNAcα1-Ser/Thr) and sT (Neu5Acα2-3Galβ1-3GalNAcα1-Ser/Thr). Their expression levels have been applied as biomarkers of poor prognosis in many cancers. Glycosylation results from the coordinated action of many factors including specific glycosyltransferases [8]. Polypeptide N-acetylgalactosaminyltransferase is a member of the family enzymes that adds N-acetylgalactosamine (GalNAc) to either threonine or serine of the polypeptide chain resulting in Tn antigen formation. The transfer of galactose (Gal) to GalNAc-1-R, by the core 1 β1,3-galactosyltransferase (C1GalT1), generates T antigen. Sialylated Tn and T antigens are formed by the GalNAc α2,6-sialyltransferase (ST6GalNAc1) and Gal α2,3-sialyltransferase (ST3Gal1), respectively [6]. Fucosylated Lewis antigens (e.g., Lewis^a^ or Lewis^b^) has been found in over 50% of cancers, including gastric ones [9]. They are synthetized by the sequential action of fucosyltransferases (FUTs) members, e.g., FUT3/4 [10].

MUC1 with cancer-associated T antigen serves as a natural ligand of galectin-3 (Gal-3), a galactose-binding protein expressed inside cells, extracellularly (cell surface associated), and in the circulation. Gal-3 reveals different biological functions, such as cell adhesion, cell–cell interaction and also RNA processing [11]. It is said that the interaction between Gal-3 and MUC1 increases cancer-cell–endothelial adhesion and, hence, promotes metastasis [12].

There is evidence about the potential anti-cancer action of natural compounds. They can participate in arresting the cell cycle, induce apoptosis, suppress cancer cell proliferation and invasiveness [13], and, cause glycosylation modifications, which is less studied. It was indicated that selected flavonoids could inhibit sialyltransferases and in this way suppress tumour metastasis [14,15]. Recently, we have suggested rosmarinic acid as anti-cancer factor by changing selected glycoforms in gastric cancer cells [16] as well as influencing apoptotic factors [17]. Luteolin has been also suggested to support anti-cancer treatment of gastric cancer by effecting MUC1 and sT antigen expression [18]. Due to a lack of reports about afzelin action in gastric cancer, we decided to investigate the effect of the flavonoid on apoptosis, the expression of MUC1, some cancer related glycoforms (as well as selected enzymes participating in their formation), and Gal-3 as factors potentially involved in malignant transformation of AGS gastric cancer cells.

## 2. Results

### 2.1. Viability of AGS Cancer Cells

**Figure 2:**
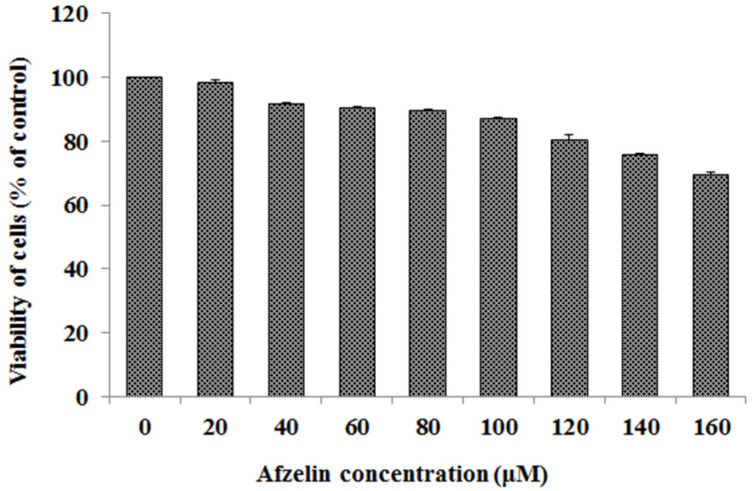
Viability of gastric cancer CRL-1739 cells (AGS) treated for 24 h with 20–160 μM concentration of afzelin. Mean values ± SD are the mean of triplicate culture.

Cell viability tests revealed low cytotoxic effect of 20–160 μM afzelin on gastric cancer cells. Viability of the cells was 70–100% compared with the control cells without afzelin (Figure 2). IC_50_ of afzelin is higher than 160 μM. In all the experiments 60 and 120 μM concentrations of the flavonoid were used.

### 2.2. Impact of Afzelin on Apoptosis

To assess the influence of afzelin on apoptosis of AGS gastric cancer cells, the Annexin V/propidium iodide double staining procedure was used. Annexin V detects phosphatidylserine exposed at the outer cell membrane and because of this, it is used to establish cells at all stages of apoptosis (Annexin V^+^/PI^−^). Late apoptosis (Annexin V^+^/PI^+^) and necrosis (Annexin V^−^/PI^+^) are identified by propidium iodide due to its staining with disrupted membranes of the cells.

**Figure 3:**
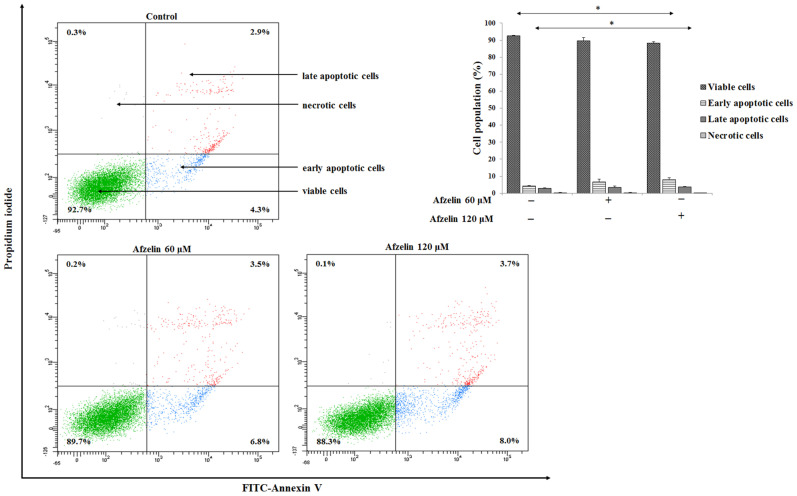
Flow cytometry determination of AGS cancer cells after 24 h incubation with 60 μM and 120 μM afzelin and subsequent staining with Annexin V and propidium iodide. The data are presented as mean percentages from 3 independent experiments done in duplicate. * *p* < 0.05, compared to the untreated control.

After 24 h of incubation with afzelin, we revealed significant induction of apoptosis upon 120 μM concentration of the flavonoid, 8% of early apoptotic cells versus 4.3% in untreated control (Figure 3).

### 2.3. The Effect of Afzelin on Bax and Caspase-3, -8, -9

**Figure 4:**
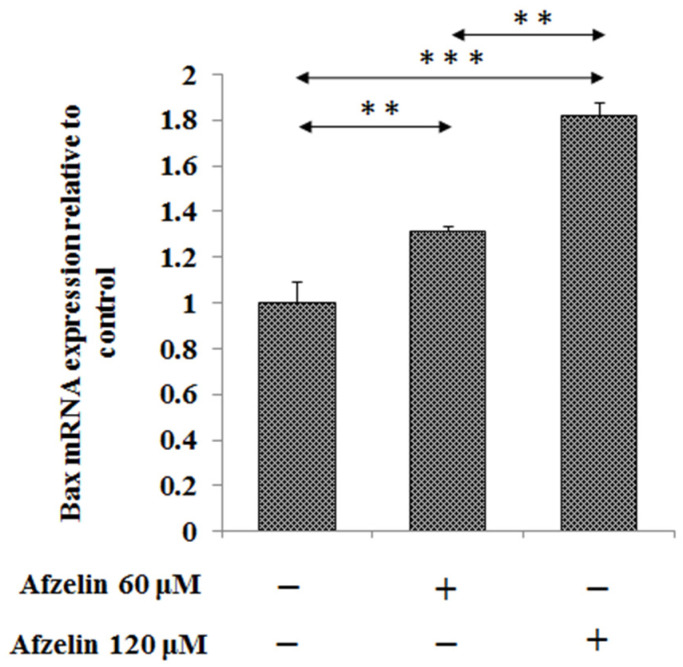
The effect of afzelin on pro-apoptotic Bax mRNA expression in AGS gastric cancer cells. The cells were incubated for 24 h with 60 and 120 μM afzelin. mRNA was determined by RT-PCR. The results are presented as a relative fold change in mRNA expression of gene in comparison of gene in control where expression was set as 1. ±SD are the mean of triplicate cultures. ** *p* < 0.01, *** *p* < 0.001.

In the next step, we assessed if afzelin initiates programmed cells death by activation of Bax pro-apoptotic factor. We revealed that Bax mRNA expression was significantly increased by 30% after incubation of the cells with 60 μM of flavonoid and by about 80% after 120 μM, in a dose dependent manner (Figure 4).

**Figure 5:**
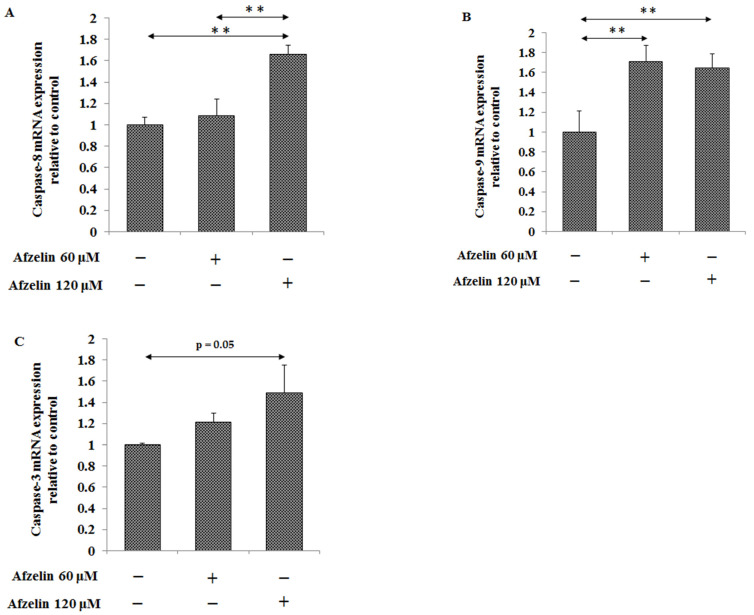
The effect of afzelin on caspase-8 (**A**), caspase-9 (**B**), and caspase-3 (**C**) mRNAs in AGS gastric cancer cells. The cells were incubated for 24 h with 60 and 120 μM afzelin. mRNA was determined by RT-PCR. The results are presented as a relative fold change in mRNA expression of gene in comparison of gene in control where expression was set as 1. ±SD are the mean of triplicate cultures. ** *p* < 0.01.

mRNAs of two initiatory caspases -8 and -9 and executive caspase-3 were determined. Caspase-8 expression was significantly stimulated, by about 60%, after 120 μM afzelin action. Lower flavonoid concentration had no effect on caspase-8 mRNA (Figure 5A). In case of caspase-9 almost the same, 60% increase of expression was observed after both afzelin doses (Figure 5B). The 120 μM afzelin also stimulated caspase-3 expression by about 50% in comparison with untreated control (Figure 5C).

### 2.4. The Effect of Afzelin on MUC1

**Figure 6:**
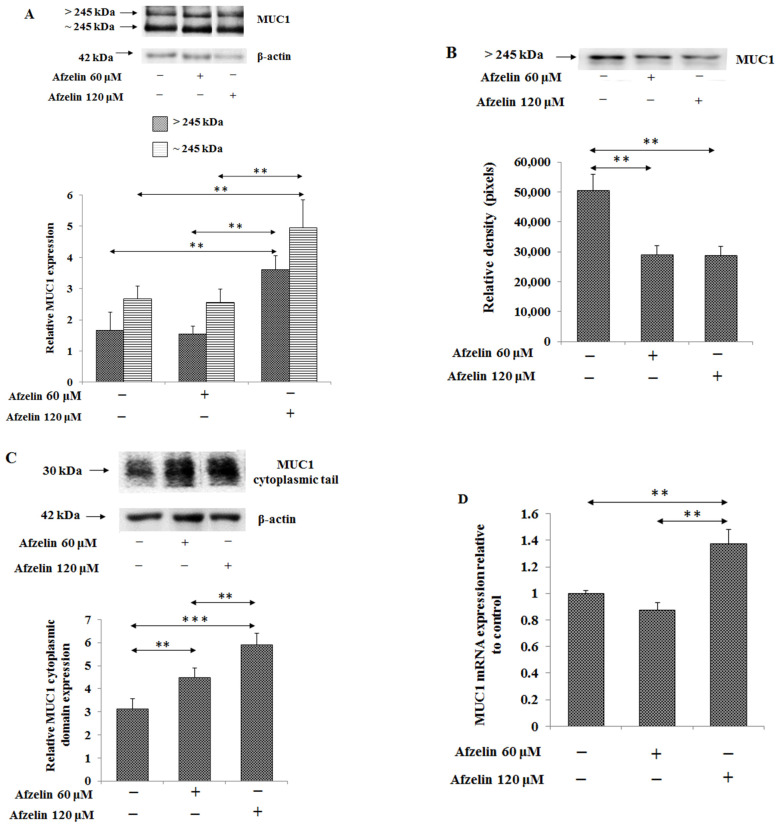
Effect of afzelin on MUC1 mucin expression in AGS gastric cancer cells treated with 60 and 120 μM afzelin. Extracellular domain expression of MUC1 was measured using Western blot analysis in cell lysates (**A**), culture medium (**B**), and cytoplasmic tail in cell lysates (**C**). β-actin expression served as protein loading control. The bands intensity was quantified by densitometry analysis. Data are presented as the mean ± SD from 3 assays. ** *p* ˂ 0.01; *** *p* ˂ 0.001. *MUC1* gene expression was determined by RT-PCR (**D**). The results are shown as a relative fold change in MUC1 mRNA in comparison to the control where expression was set as 1. ±SD are the mean of triplicate cultures. ** *p* ˂ 0.01.

The extracellular domain of MUC1 mucin present in gastric cancer cell lysates and culture medium was differently affected by afzelin. The flavonoid with 120 μM concentration significantly increased expression of two extracellular MUC1 subunits in cell lysates: subunit with ~245 kDa molecular mass increased by over 100% and band higher than 245 kDa increased by about 80% compared to untreated cells (Figure 6A, supplementary Figure S1). The expression of the over 245 kDa extracellular mucin domain released to the culture medium revealed a significant decrease, about 42%, after treatment of the cells with both concentrations of the flavonoid in comparison to control (Figure 6B, supplementary Figure S1). Cytoplasmic domain of MUC1 with 30 kDa molecular mass was significantly increased by afzelin treatment, in concentration dependent manner by 43 and 88%, respectively (Figure 6C, supplementary Figure S1). It was also found that 120 μM afzelin exerted by 37% stimulatory effect on MUC1 mRNA expression (Figure 6D).

### 2.5. The Effect of Afzelin on Tn, T, sTn, and sT Antigens

**Figure 7:**
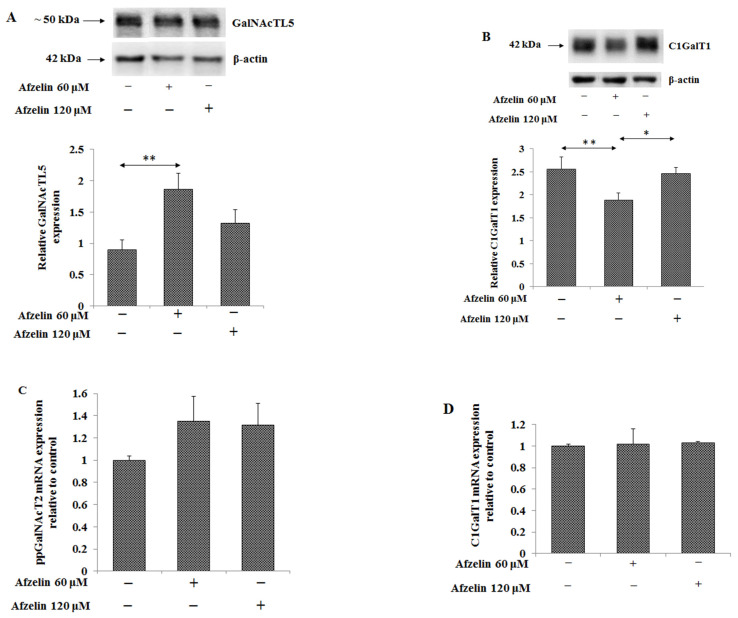
Effect of afzelin on the expression of enzymes taking part in Tn (GalNAcα1-O-Ser/Thr) and T (Galβ1-3GalNAcα1-O-Ser/Thr) antigens formation in AGS gastric cancer cells treated with 60 and 120 μM afzelin. Expression of GalNAcTl5 (**A**) and C1GalT1 (**B**) in cell lysates was measured using Western blot analysis. The β-actin expression served as protein loading control. The bands intensity was quantified by densitometry. Data are presented as the mean ± SD from 3 assays. * *p* ˂ 0.05; ** *p* ˂ 0.01; *ppGalNAcT2* (**C**) and *C1GalT1* (**D**) gene expressions were determined by RT-PCR. The results are shown as a relative fold change in ppGalNAcT2 and C1GalT1 mRNA in comparison to control where expression was set as 1. ±SD are the mean of triplicate cultures.

Tn and T antigens are typical carbohydrate cancer antigens present on MUC1. GalNAcTL5 is the enzyme participating in Tn antigen formation at the initiation of O-linked glycosylation. In Figure 7A (and supplementary Figure S2) stimulatory effect of 60 μM afzelin, by about 100%, on GalNAcTL5 protein expression is presented. T antigen is formed by Gal addition to Tn antigen in the reaction catalyzed by C1GalT1. 60 μM afzelin significantly inhibited the enzyme protein expression by 27%. (Figure 7B, supplementary Figure S2). The expression of enzymes responsible for Tn and T antigens formation was also checked on the mRNA level. However, no significant effect of afzelin on ppGalNAcT2 and C1GalT1 mRNAs was observed (Figure 7C,D).

**Figure 8:**
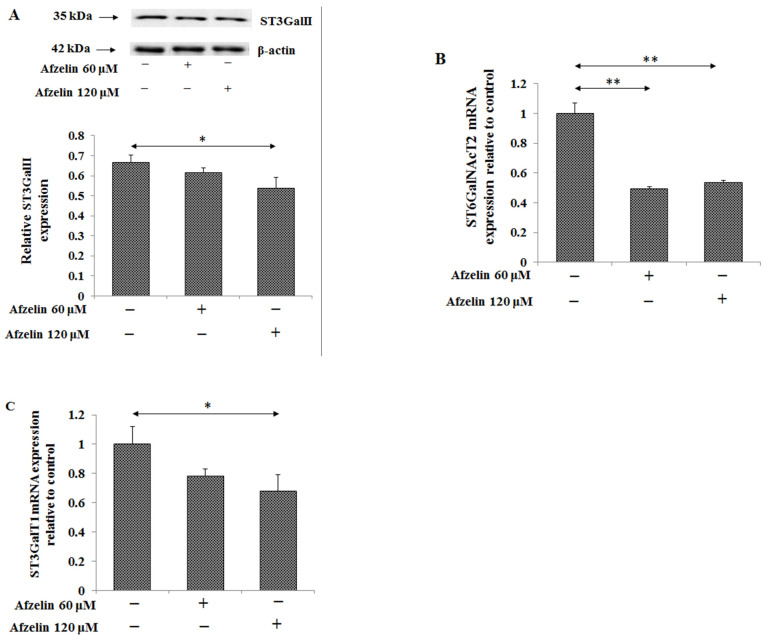
Effect of afzelin on the expression of enzymes participating in sialyl Tn and sialyl T antigens formation in AGS gastric cancer cells treated with 60 or 120 μM afzelin. Expression of ST3GalII (**A**) in cell lysates was measured using Western blot analysis. The β-actin expression served as protein loading control. The bands intensity was quantified by densitometry. Data are presented as the mean ± SD from 3 assays. * *p* ˂ 0.05; *ST6GalNAcT2* (**B**) and *ST3GalT1* (**C**) gene expressions were assessed by RT-PCR. The results are expressed as a relative fold change in ST6GalNAcT2 and ST3GalT1mRNA expression in comparison to control where expression was set as 1. ±SD are the mean of triplicate cultures. * *p* ˂ 0.05; ** *p* ˂ 0.01.

Sialyl Tn and T antigens are other common cancer related structures. Sialic acid can be linked to Gal/GalNAc by α2-6 bond or by α2-3 bond in sialyl Tn and sialyl T antigens, respectively. ST6GalNAcT2 is sialyltransferase that transfers sialic acid onto Tn antigen and ST3GalT1 is enzyme responsible for sialyl T antigen formation. Western blotting analysis revealed a significant, by 20%, inhibitory effect of 120 μM afzelin on ST3GalII protein (Figure 8A, supplementary Figure S3) as well as by about 50% on *ST6GalNAcT2* with both afzelin concentrations and by 32% on *ST3GalT1* with 120 μM afzelin (Figure 8B and Figure 8C, respectively). The expression of ST6GalNAcT on the protein level was not examined.

### 2.6. The Effect of Afzelin on Lewis Antigens

**Figure 9:**
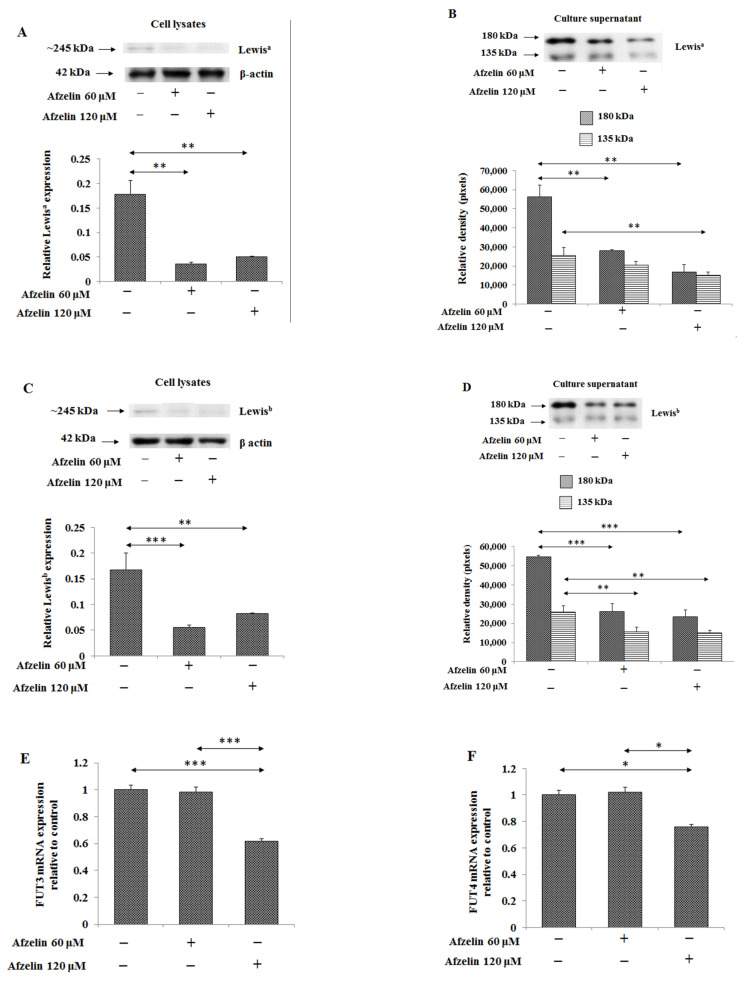
Effect of afzelin on Lewis^a^ and Lewis^b^ antigen expression as well as enzymes participating in these antigens’ formation in AGS gastric cancer cells treated with 60 and 120 μM afzelin. Western blotting was used to determine Lewis^a^ (**A**,**B**) and Lewis^b^ (**C**,**D**) structures in cell lysates and culture medium. The β-actin expression served as protein loading control. The bands intensity was quantified by densitometry. Data are presented as the mean ± SD from 3 assays. * *p* ˂ 0.05; ** *p* ˂ 0.01; *** *p* ˂ 0.001. *FUT3* (**E**) and *FUT4* (**F**) gene expressions were assessed by RT-PCR. The results are shown as a relative fold change in FUT3 and FUT4 mRNA in comparison to control where expression was set as 1. ±SD are the mean of triplicate cultures. * *p* ˂ 0.05; *** *p* ˂ 0.001.

Lewis^a^ (Galβ1-3(Fucα1-4)GlcNAcβ-R) and Lewis^b^ ((Fucα1-2)Galβ1-3(Fucα1-4)GlcNAcβ-R) are structures notably associated with cancers, present especially at terminal positions of oligosaccharide chains. In cell lysates, Lewis^a^ antigen expression was significantly decreased, by 80 and 70%, after both concentrations of afzelin treatment, and Lewis^b^ antigen by 67 and 51%. In the culture medium, for Lewis^a^, expression decreased by 70–40% and by 41–57% for Lewis^b^ (Figure 9A–D, supplementary Figure S4). FUT3 and FUT4 are fucosyltransferases representing α1,3 and α1,4-fucosyltransferase activity. An amount of 120 μM of afzelin significantly inhibited *FUT3* and *FUT4* gene expressions by 38% and 24% (Figure 9E and Figure 9F, respectively).

### 2.7. The Effect of Afzelin on Gal-3

**Figure 10:**
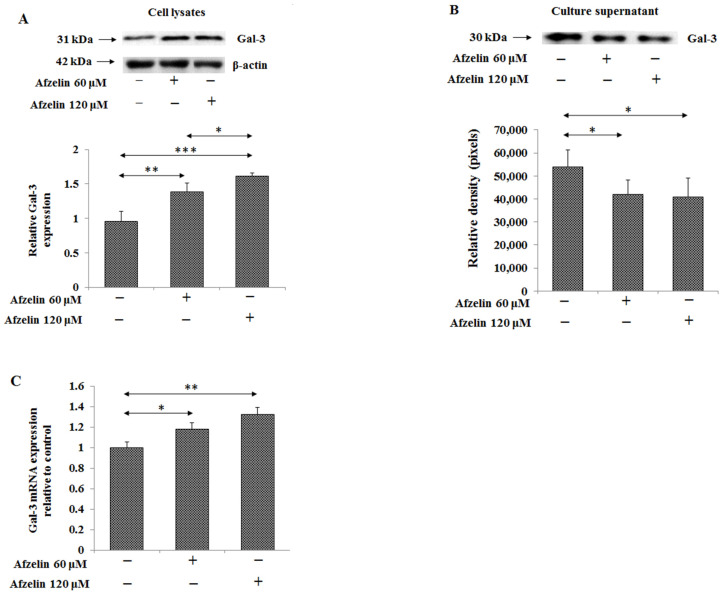
Effect of afzelin on Gal-3 in AGS gastric cancer cells treated with 60 and 120 μM afzelin. Western blotting was used to determine Gal-3 in cell lysates (**A**) and culture medium (**B**). The β-actin expression served as protein loading control. The bands intensity was quantified by densitometry. Data are presented as the mean ± SD from 3 assays. * *p* ˂ 0.05; ** *p* ˂ 0.01; *** *p* ˂ 0.001. *Gal-3* gene expression (**C**) was assessed by RT-PCR. The results are shown as a relative fold change in Gal-3 mRNA expression in comparison to control where expression was set as 1. ±SD are the mean of triplicate cultures. * *p* ˂ 0.05; ** *p* ˂ 0.01.

Gal-3 is β-galactoside-binding protein, distributed inside and outside of the cell. MUC1 mucin with TF antigen can be one of possible ligands for Gal-3. In the current study, a significant, concentration dependent increase of Gal-3 expression in cells lysates was revealed, by 45 and 69% for 60 and 120 μM afzelin, respectively (Figure 10A, supplementary Figure S5). On the contrary, decreased expression of Gal-3 protein in culture medium by about 30% without relation to flavonoid concentration was observed (Figure 10B, supplementary Figure S5). Gal-3 mRNA was significantly stimulated by 17% after 60 μM and by 32% after 120 μM afzelin treatment (Figure 10C).

## 3. Discussion

Many flavonoids exert anti-cancer activity such as: modulation ROS-scavenging enzyme activities, induction of apoptosis, autophagy or suppression of proliferation and invasiveness. However, the mechanisms of their action have not been thoroughly clarified yet [13]. They seem to be beneficial due to their abundancy in the diet and generally low side effects while using them during treatment [19]. Afzelin is an example of such compounds. According to our knowledge, there are no studies about afzelin action in gastric cancer, thus, we decided to check how it influences several cancer related factors in AGS gastric cancer cells.

Apoptosis-inducing therapy is said to be very desirable, safe, and effective anti-cancer approach, revealing also by natural compounds [13,20]. Recently we have demonstrated stimulation of apoptotic response by rosmarinic acid in gastric cancer cells [17]. The acid affected pro- and anti-apoptotic factors as well as caspases. Such an action of natural product was also revealed in the present study, as afzelin induced apoptosis by increasing the expression of pro-apoptotic *Bax* and *caspases*.

It is well established that glycans are involved in various steps of cancer development. They participate in cell–cell and cell–matrix interactions, cell differentiation, migration, invasion, and metastasis. Many epithelial cancers are associated with alterations in MUC1 expression and its glycosylation what affects a variety of cellular activities [6]. Cytoplasmic tail of MUC1 was demonstrated to be applied in intracellular signal transduction, e.g., by association with some transcription factors (e.g., NF-κB, p53, β-catenin) and modulation of their function. Aberrant glycosylation of extracellular part of MUC1 can stimulate its endocytosis and intracellular accumulation what can alter its general role in intracellular signalling [7]. MUC1 protein can attach to intercellular adhesion molecule-1 (ICAM-1), which promotes breast cancer cells adhesion to endothelial cells, leading to adhesion and consequent migration through the vessel wall [21]. It was also reported that MUC1 extracellular domain prevents initiation of anoikis, apoptosis occurring in replay to loss of cell adhesion to the extracellular matrix [22]. In our study we revealed stimulatory effect of afzelin on mucin cytoplasmic tail as well as on *MUC1* gene expression. It is said that cytoplasmic tail of the mucin is identical in normal and tumour cells and the greatest difference between normal and malignant cells is in MUC1 localization [23]. However, in this study, the exact cellular localization of MUC1 as well as the expression of specific protein factors potentially interacting with MUC1 cytoplasmic tail in modulations of cell signalling pathways were not analysed. Thus, upon our results it is difficult to assess the role of afzelin in regard to intracellular part of MUC1. Interestingly, our outcomes concerning MUC1 mRNA and its cytoplasmic tail expression are not in agreement with the results received by Zhou et al. [24]. The authors demonstrated flavone apigenin as the product inhibiting *MUC1* gene and MUC1 cytoplasmic domain expression in cancer. Recently, we also demonstrated rosmarinic acid suppressing MUC1 mRNA as well as MUC-1 cytoplasmic tail in gastric cancer cells [17]. We suggest that to explain such discrepancy extra experiments are needed to be performed. However, we can postulate different mechanisms of action of the mentioned flavonoids. We demonstrated the inhibitory action of afzelin on MUC1 extracellular domain released to the culture medium. This result is in consistence with recently reported, inhibitory action of quercetin on MUC1 in breast cancer cells. The authors concluded that such action of flavonoid could be correlated with decreased proliferation and metastasis as specific, sugar cancer-related antigens, present on MUC1, were reduced [25].

Tumour glycans consist both relatively short and very extended structures, involving truncated glycans such as Tn, T antigens, and their sialylated sTn and T forms, which are said to be tumour specific (present especially in tumours), as well as the extended glycans with fucosylated Lewis antigens, which are said to be tumour-associated (found in tumours but present also in some normal tissues) [26]. Such altered glycosylation facilitates, e.g., MUC1, main carrier of mentioned antigens in endothelial cells, to function as a ligand for, e.g., cell adhesion molecules and seeding at distant sites that forms secondary tumours [7]. Glycosylation during carcinogenesis can be altered by the modified expression and localization of specific glycosyltransferases. GalNAc transferases, enzymes initiating the mucin-type O-glycosylation, crucial regulatory step, are often changed in cancers [26]. High expression of ppGalNAcT2 was demonstrated to inhibit the metastatic ability of SGC7901 human gastric cancer cells [27]. It was also noted that downregulation of ppGalNAcT2 increased the cancer cell proliferation, adhesion and invasion in gastric cancer cells [28]. C1GalT1, an enzyme participating in the elongation of Tn structure, is often overexpressed in tumours, causing accumulation of T antigens [8]. It was reported that excessive expression of C1GalT1 in colorectal tumour tissues was associated with invasion, metastasis, and poor survival [29]. Similar findings connecting C1GalT1 overexpression and enhanced malignant growth of breast cancer were demonstrated by Chou et al. [30]. Thus, we postulate that stimulatory effect of afzelin on GalNAcTL5 protein expression and inhibition of C1GalT1 in comparison to untreated control can be understood as potential anti-cancer action of the flavonoid, participating in metastasis prevention.

Hypersialylation of glycoproteins on cell surfaces correlates with lowered cancer cell adhesion, extended cancer cell invasion, and poor prognosis [31]. Sialyl Tn antigen in gastric cancer cells was indicated to generate a more aggressive cell behaviour by, e.g., promoting lowered cell–cell aggregation, altered ECM adhesion, and increased migration and invasion in vitro [32]. Examples of enzymes participating in sialyl Tn and sialyl T antigens formation are ST6GalNAcT2 and ST3GalT, respectively. Inhibitory action of afzelin on ST6GalNAcT2 mRNA, ST3GalT1 mRNA and ST3GalII protein expression, revealed in our study can suggest the flavonoid as the factor potentially changing cell–cell, cell-ECM interactions resulting potentially in decreasing of cells migration.

Elevated expression of FUTs and fucosylated carbohydrate structures during malignant cell transformation are in relation with the recruit of an increased proliferative potential and also with pro-survival phenotype [21,33]. In some cases, suitability of fucosylated epitopes for cancer management has been utilized, e.g., fucosylated α-fetoprotein has been established as a marker for early detection of hepatocellular carcinoma; fucosylated haptoglobin has been also approved as biomarker for pancreatic and colon cancer [34,35]. In the present work, analysis of Lewis^a^ and Lewis^b^ antigens level as well as FUT3/4 mRNA demonstrated inhibitory effect of afzelin on mentioned factors in comparison with untreated control. In our opinion, these results may also suggest usefulness of afzelin as prospective anti-cancer agent suppressing cancer development.

The last factor analysed in our study was Gal-3. Its extracellular location associates with cell attachment, angiogenesis, cell proliferation, promoting cancer progression, and metastasis [11,12,31]. It has been stated that MUC1, with T antigen is natural ligand for Gal-3, and the interaction between galectin and cancer-associated MUC1 enhances adhesion of cancer cells to each other and to endothelial cells and, hence, promotes metastasis [6,12]. It was recently reported that morin (isomer of quercetin) decreased the expression of Gal-3 in ovarian cancer cells what sensitized the cancer cells to cisplatin [36]. Concerning extracellular Gal-3 level, afzelin seems to act as an anti-tumour agent as decreased expression of galectin upon flavonoid action was observed. This result correlated with extracellular expression of MUC1. Less is known about the role of intracellular Gal-3. It was reported that in the cytoplasm, galectin seems to be important for cell survival, e.g., due to interaction with pro-survival Bcl-2, whereas in the nucleus Gal-3 promotes pre-mRNA splicing and controls gene transcription. It was demonstrated that in 50% of examined gastric tumours, tissue Gal-3 expression was reduced by 1.5-fold. This reduced Gal-3 expression level was correlated with the distant metastasis, and with a higher invasive phenotype in vitro [10]. Stimulatory action of afzelin on mRNA and relative Gal-3 expression in cell lysates of gastric cancer cells revealed in our study seems to be one more proof of potential utilizing of afzelin in anti-cancer treatment.

## 4. Materials and Methods

### 4.1. Cell Culture

Human gastric adenocarcinoma cells CRL-1739 (AGS) were obtained from ATCC (Manassas, VA, USA) and kept in F-12 medium (Gibco, Waltham, MA, USA) enriched with streptomycin (100 μg/mL), penicillin (100 U/mL) (Sigma, St. Louis, MO, USA), and Fetal Bovine Serum (FBS) (10%) (Gibco, Waltham, MA, USA) at 37 °C in a humidified atmosphere of 5% CO_2_. In the next step the cells were seeded in 6-well plates (5 × 10^5^ cells/well) and cultivated for 24 h in medium (FBS-free) supplemented with 60 and 120 μM afzelin (≥90% (LC/MS-UV) (Sigma, St. Louis, MO, USA). Stock solution of afzelin was 50 mM (2 mg /50 μL DMSO; Sigma, St. Louis, MO, USA). After washing the wells (Phosphate Buffered Saline, pH 7.4 (PBS)), the cells were lysed 20 min at 4 °C with RIPA buffer (Sigma, St. Louis, MO, USA) containing protease inhibitors (Sigma, St. Louis, MO, USA), diluted in RIPA buffer (1:200). Collected lysates and culture media were centrifuged at 1000× *g* for 5 min at 4 °C. The obtained supernatants were frozen at −70 °C and then used for Western blot assays. For RT-PCR the monolayers were washed with sterile 10 mM PBS (three times) and sonicated (Sonics Vibra cell; Sonics & Materials, Leicestershire, UK). Aliquots of the homogenate were applied for RNA isolation. Cells without afzelin addition were used as control.

### 4.2. Cell Viability Test

Cell viability test was performed with 3-(4,5-dimethylthiazole-2-yl)-2,5-diphenyltetrazolium bromide; MTT (Sigma, St. Louis, MO, USA), in accordance with the protocol of Carmichael et al. [37]. After 24 h cell culture with 20–160 μM afzelin concentrations in 6-well plates, 1 mL of MTT solution (0.5 mg of MTT/mL of PBS) was added to each well and incubated at 37 °C in a 5% CO_2_ incubator for 4 h. Absorbance of converted dye in living cells was read at 570 nm. Viability of AGS cells treated with afzelin was considered as a percentage of the cells without afzelin (negative control with 100% cell viability).

### 4.3. Flow Cytometry Assessment of Annexin V Binding

To evaluate the mode of cell death induced by afzelin, a flow cytometry measurement was performed using Apoptosis Detection Kit II (BD Pharmingen, San Diego, CA, USA), according to the manufacturer’s instructions. Cells were trypsinised, resuspended in the medium, and then in the binding buffer. In the next step, they were stained with Annexin V and propidium iodide (PI) 15 min at room temperature (RT) in the dark place. As a negative control, the cells cultured in a drug-free medium were used. Optimal parameter settings were found using a positive control—cells incubated with 3% formaldehyde in buffer, for 30 min on ice. To analyse the results, FACSCanto II cytometer (BD, San Jose, CA, USA) with FACSDiva software (BD Biosciences systems, San Jose, CA, USA) was applied.

### 4.4. Western Blotting

The samples were mixed with probe sample buffer (4:1) supplemented with SDS (2.5%) (Sigma, St. Louis, MO, USA). The same protein aliquots (20 μg) of lysates or equal volumes of concentrated medium were separated on 7.5 or 10% polyacrylamide gels and then immunoblotted on an Immobilon P membrane (Millipore, Bedford, MA, USA) in accordance the procedure described previously [16]. Used antibodies are specified in Table 1. Enhanced chemiluminescence with Westar Supernova, ECL substrate for Western blotting (Cyangen, Bologna, Italy) was applied to detect bound antibodies. The intensities of the bands were determined by densitometric analysis. The β-actin expression served as protein loading control.

### 4.5. Real-Time PCR

Total RNA Mini Plus Concentrator (A&A Biotechnology, Gdynia, Poland) was applied to extract total RNA. Purity and concentration of RNA was determined spectrophotometrically (Nanodrop 2000, Thermo Scientific, Waltham, MA, USA). Subsequently, first-strand cDNA was synthesized from 1 μg of total RNA applying Tetro cDNA Synthesis Kit (Bioline, London, UK). A total of 20 μL of the reaction mixture with 1 μL oligo(dT)_18_ primer, 1 μL of dNTP mixture (10 mM each), 5 μL of 5 × RT Buffer, 1 μL of RiboSafe RNase Inhibitor (10u/μL), 1 μL of Tetro Reverse Transcriptase (200 u/μL), and DEPC-treated water was kept for 30 min at 45 °C and then incubated at 85 °C for 5 min. RT-PCR assay was carried out in CFX96 Real-time instrument (Bio-Rad, Hercules, CA, USA) using SensiFAST^TM^ SYBR Kit (Bioline, London, UK). The reactions consisted of 2 μL of cDNA template (2-times diluted), 0.8 μL of each primer (10 μM), 10 μL 2 × SensiFAST SYBR Mix and nuclease-free water (in 20 μL of a final volume). Primers (Table 2) were synthesized by Genomed (Warsaw, Poland). Glyceraldehyde-3-phosphate dehydrogenase (*GAPDH*) was evaluated as housekeeping gene. The program parameters of PCR were as follows: 1 min at 95 °C to activate the DNA polymerase, then 40 cycles of 10 s at 95 °C, 15 s at 60 °C, 20 s at 72 °C. To confirm the specificity of the amplified products, the reaction was exposed to a melting protocol from 55 °C to 95 °C with a 0.2 °C increment and 1 s holding at each increment. Single product production was proved by melting point assessment and agarose gel electrophoresis (water, instead of mRNA, samples was used as negative control). Samples were run in triplicate and, for data analysis, relative expression was determined with the ΔΔCT method.

### 4.6. Statistical Analysis

Experimental data were presented as mean ± standard deviation SD from at least three independent experiments. One-way ANOVA test followed by the Duncan’s multiple range post hock was applied for determination of statistical differences. All statistical analysis was performed using Statistica package (StatSoft, Tulsa, OK, USA). *p* values less than 0.05 was considered to be significant.

## 5. Conclusions

The present work, for the first time, demonstrates the potential anti-cancer action of afzelin in gastric cancer cells. This general conclusion is based on the following outcomes: (a.) afzelin stimulated apoptotic respond likely by inducing the expression of pro-apoptotic Bax as well as caspase-8,-9,-3 mRNAs; (b.) the flavonoid decreased the expression of MUC1 extracellular domain as well as extracellular expression of Gal-3. Assuming that MUC1 serves as primary carrier of T antigen, the main ligand for Gal-3, we postulate that afzelin can be involved in potential suppression of metastasis; (c.) afzelin inhibited C1GalT1 protein expression as well as ST6GalNAcT2, ST3GalT1 mRNAs, and ST3GalII protein responsible for sialylation of Tn and T antigens. Decreased sialylation contributed to lowered invasiveness of cancer cells; and (d.) the flavonoid suppressed fucosylation, one more factor connecting to proliferative potential of cancer cells.

We want to state that we are aware of limitations of our study. There are points of our work, especially concerning the mechanism of the flavonoid action on gastric cancer cells, that should be elucidated in the future. In vivo studies are also required. However, we assume that afzelin could be considered as a natural product supporting gastric cancer therapy.

## Figures and Tables

**Table 1:** Source of antibodies.

Antibody	Clone	Source
Anti-MUC1; extracellular domain (mouse IgG) Anti-MUC1; cytoplasmic tail (Armenian hamster IgG) Anti-Lewis^a^ (mouse IgG) Anti-Lewis^b^ (mouse IgG) Anti-C1GalT1 (mouse IgG) Anti-ST3GalII (mouse IgG) Anti-GalNAcTL5 (mouse IgG) Anti-Gal-3 (mouse IgG)	BC2 CT2 7LE LWB01 F-31 34-K F-5 B2C10	Abcam Abcam Santa Cruz Thermo Scientific Santa Cruz Santa Cruz Santa Cruz Santa Cruz
Anti-β-actin (rabbit IgG) Anti-mouse IgG peroxidase conjugated Anti-rabbit IgG peroxidase conjugated Anti-Armenian hamster IgG peroxidase conjugated		Sigma Sigma Sigma Abcam

**Table 2:** Sequences of primers used for RT-PCR.

Gene	Forward Primer (5′ → 3′)	Reverse Primer (5′ → 3′)
*Bax*	TTGCTTCAGGGTTTCATCCA	CAGCCTTGAGCACCAGTTTG
*Caspase-3*	CAGTGGAGGCCGACTTCTTG	TGGCACAAAGCGACTGGAT
*Caspase-8*	TTTCTGCTGAAGTCCATCTTTTT	TAGGGGACTCGGAGACTGC
*Caspase-9*	CCCATATGATCGAGGACATCCA	ACAACTTTGCTGCTTGCCTGTTAG
*MUC1*	TGCCTTGGCTGTCTGTCAGT	GTAGGTATCCCGGGCTGGAA
*C1GalT1*	AAGCAGGGCTACATGAGTGG	GCATCTCCCCAGTGCTAAGT
*ppGalNAcT2*	AAGAAAGACCTTCATCACAGCAATGGAGAA	ATCAAAACCGCCCTTCAAGTCAGCA
*ST6GalNAcT2*	CCTTCTGAACGGCTCAGAGAGT	GCACACCGGATACACTTTGGA
*ST3GalT1*	TCGGCCTGGTTCGATGA	CGCGTTCTGGGCAGTCA
*FUT3*	GCCGACCGCAAGGTGTAC	TGACTTAGGGTTGGACATGATATCC
*FUT4*	AAGCCGTTGAGGCGGTTT	ACAGTTGTGTATGAGATTTGGAAGCT
*Gal-3*	GCAGACAATTTTTCGCTCCATG	CTGTTGTTCTCATTGAAGCGTG
*GAPDH*	GTGAACCATGAGAAGTATGACAA	CATGAGTCCTTCCACGATAC

## Data Availability

Data is contained within the article and supplementary material.

## Supplementary Materials

The following are available online at https://www.mdpi.com/article/10.3390/ph14100973/s1, Figure S1: Original images of Western blots to Figure 6A–C; Figure S2: Original images of Western blots to Figure 7A,B; Figure S3: Original images of Western blots to Figure 8A; Figure S4: Original images of Western blots to Figure 9A–D; Figure S5: Original images of Western blots to Figure 10A,B.
