# Supplementary figures and images for: Anti-Cancer Potential of Afzelin towards AGS Gastric Cancer Cells
Source: Pharmaceuticals (Basel). 2021 Sep 25;14(10):973. doi: 10.3390/ph14100973 (PMC8539446; doi:10.3390/ph14100973)

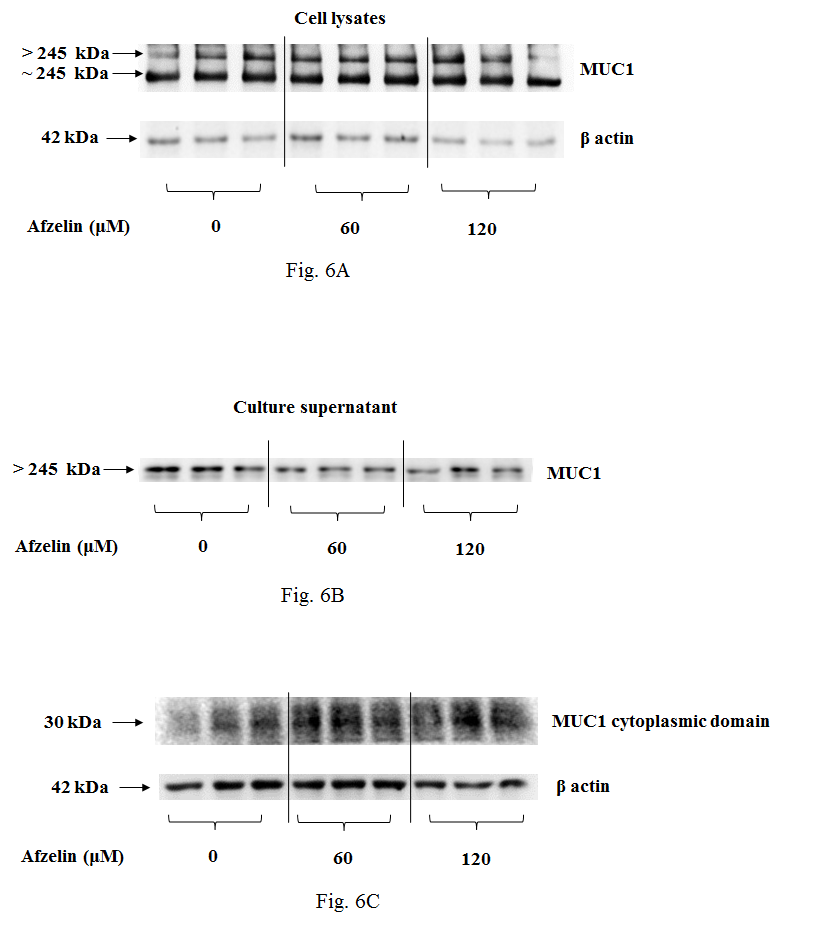

Supplement: Supplementary file 1 [file pharmaceuticals-14-00973-s001.zip › Figure S1.tif]

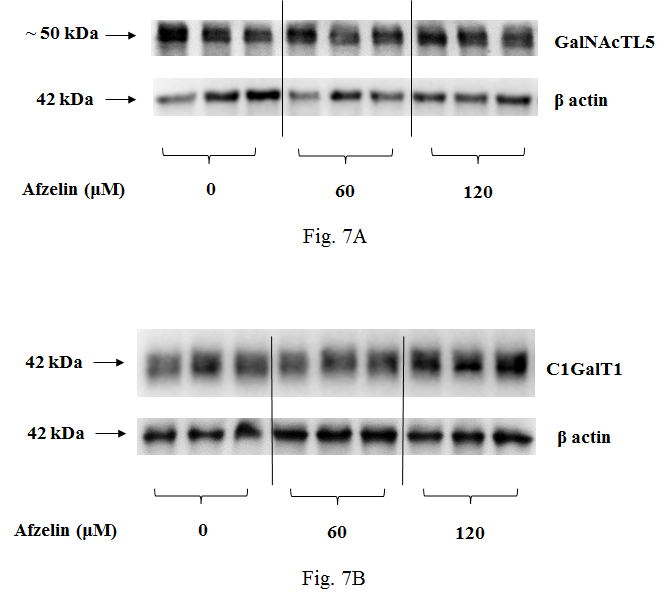

Supplement: Supplementary file 1 [file pharmaceuticals-14-00973-s001.zip › Figure S2.tif]

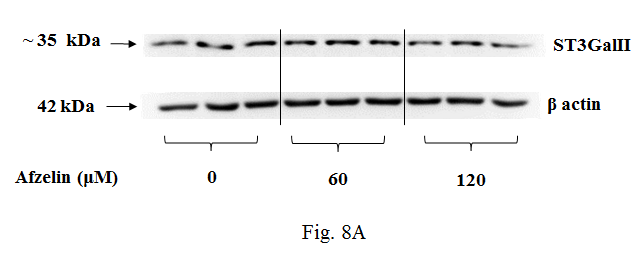

Supplement: Supplementary file 1 [file pharmaceuticals-14-00973-s001.zip › Figure S3.tif]

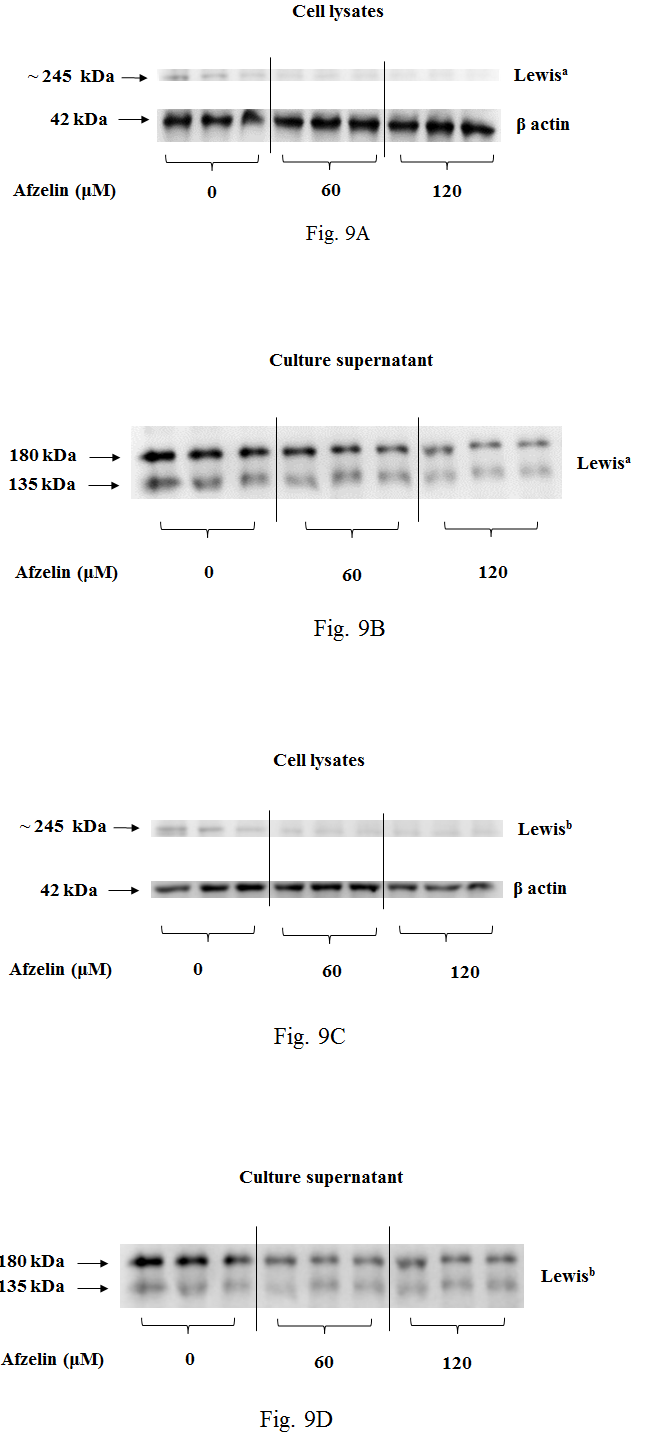

Supplement: Supplementary file 1 [file pharmaceuticals-14-00973-s001.zip › Figure S4.tif]

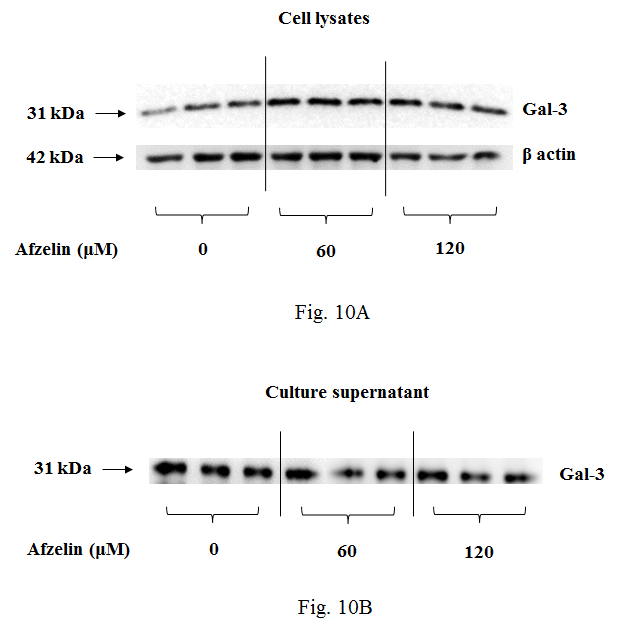

Supplement: Supplementary file 1 [file pharmaceuticals-14-00973-s001.zip › Figure S5.tif]
